# Larval habitat diversity and *Anopheles* mosquito species distribution in different ecological zones in Ghana

**DOI:** 10.1186/s13071-021-04701-w

**Published:** 2021-04-07

**Authors:** Isaac A. Hinne, Simon K. Attah, Benedicta A. Mensah, Akua O. Forson, Yaw A. Afrane

**Affiliations:** 1 Department of Medical Microbiology, University of Ghana Medical School, University of Ghana, Korle-Bu, Accra, Ghana; 2 Department of Epidemiology, Noguchi Memorial Institute of Medical Research, College of Health Sciences, University of Ghana, Legon, Ghana; 3 Department of Medical Laboratory Science, School of Biomedical and Allied Health Sciences, University of Ghana, Korle-Bu, Accra, Ghana

**Keywords:** *Anopheles*, Larval habitats, Larval abundance, Ecological zones, Ghana

## Abstract

**Background:**

Understanding the ecology of larval malaria and lymphatic filariasis mosquitoes in a changing environment is important in developing effective control tools or programmes. This study characterized the breeding habitats of *Anopheles* mosquitoes in rural communities in different ecological zones in Ghana during the dry and rainy seasons.

**Methods:**

The spatio-temporal distribution, species composition, and abundance of larval *Anopheles* mosquitoes in breeding habitats were studied in five locations in three ecological zones of Ghana. These were Anyakpor (coastal savannah area), Duase (forest area), and Libga, Pagaza, and Kpalsogu (Sahel savannah area). Larvae were collected using standard dippers and were raised in the insectary for identification.

**Results:**

Out of a total of 7984 mosquito larvae collected, 2152 (27.26%) were anophelines and were more abundant in the rainy season (70.82%) than in the dry season (29.18%). The anophelines comprised 2128 (98.88%) *An. gambiae* s.l., 16 (0.74%) *An. rufipes*, and 8 (0.37%) *An. pharoensis*. In the coastal savannah and forest zones, dug-out wells were the most productive habitat during the dry (1.59 larvae/dip and 1.47 larvae/dip) and rainy seasons (11.28 larvae/dip and 2.05 larvae/dip). Swamps and furrows were the most productive habitats in the Sahel savannah zone during the dry (0.25 larvae/dip) and rainy (2.14 larvae/dip) seasons, respectively. *Anopheles coluzzii* was the most abundant sibling species in all the ecological zones. *Anopheles melas* and *An. arabiensis* were encountered only in the coastal savannah and the Sahel savannah areas, respectively. Larval habitat types influenced the presence of larvae as well as larval density (*p* < 0.001). The land-use type affected the presence of *Anopheles* larvae (*p* = 0.001), while vegetation cover influenced larval density (*p* < 0.05).

**Conclusion:**

The most productive habitats were dug-out wells in the coastal savannah and forest zones, and furrows from irrigated canals in the Sahel savannah zone. *Anopheles coluzzii* was the predominant vector species in all the ecological zones. The abundance of *Anopheles* breeding habitats and larvae were influenced by anthropogenic activities. Encouraging people whose activities create the larval habitats to become involved in larval source management such as habitat manipulation to stop mosquito breeding will be important for malaria and lymphatic filariasis control.

**Supplementary Information:**

The online version contains supplementary material available at 10.1186/s13071-021-04701-w.

## Background

*Anopheles* mosquitoes are important vectors that transmit diseases including malaria and lymphatic filariasis, among others [1]. The distribution and abundance of adult *Anopheles* mosquitoes are predicated on the presence and productivity of larval breeding habitats [2]. Species of the *Anopheles gambiae* complex prefer to breed in shallow water collections that are open to sunlight [3]. Their breeding habitats can have varying sizes of water bodies that are natural or man-made, permanent or temporary, freshwater or saline [2, 4]. *Anopheles funestus*, on the other hand, prefer to breed in shady permanent or semi-permanent water bodies, usually with floating or emergent vegetation such as that found in swamps, marshes, and edges of streams [5, 6].

The choice of oviposition sites of mosquitoes is influenced by myriad environmental factors, which include climatic components such as temperature, rainfall, vegetation, salinity and turbidity of the water, the size of the habitat, and the amount of sunlight [2]. The temperature of larval habitats can influence larval development, pupation rate and time, and larval survivorship [2, 7]. Variation in rainfall patterns or seasonal changes can also affect the availability of larval habitats and larval productivity [3].

Vector control is key to the elimination of vector-borne diseases such as malaria and lymphatic filariasis [8, 9]. Even though the most widely used vector control methods—long-lasting insecticide nets (LLINs) and indoor residual spraying (IRS)—have reduced the transmission of malaria in Africa [10, 11], these methods have not been successful in malaria eradication because of the emergence and rapid spread of insecticide resistance in mosquitoes [12–14]. Also, the use of LLINs and IRS which target indoor-biting and indoor-resting mosquitoes have driven behavioural changes in the *Anopheles* mosquito from indoor, late-night biting to early biting times when humans might be unprotected outside [15, 16]. Nevertheless, larval source management or source control could provide an additional valuable tool for the control of malaria vectors [17]. To assess the feasibility of larval control or larval source management, there is the need to assess the abundance of different types of habitats, measure the productivity in each habitat type [18], and also understand how these different habitat types are formed and how they interact with the society.

The presence and density of mosquito larvae, and consequently the number of competent adult malaria vectors, are regulated by a variety of ecosystem processes interacting at different levels and spatio-temporal scales. These include the presence of water and aquatic plants that protect larvae from predators and serve as detritus that support microbial communities, which in turn serve as food for mosquito larvae [19]. Changes in the structure of the ecosystem can have a considerable impact on mosquito populations and species distribution. Therefore, studies on the ecology of larval habitats should include a landscape context [2, 3, 20, 21]. Landscape features such as topography, land cover, and land use influence the formation, distribution, and microclimate conditions of larval habitats, which in turn influence the distribution of adult *Anopheles* vectors [2, 22–25]. Human activities can affect habitat distribution and stability through landscape changes such as deforestation, irrigation, and agricultural practices [2].

There are three main ecological zones in Ghana—the coastal savannah in southern Ghana, the forest in central Ghana, and the Sahel savannah in northern Ghana. These ecological zones affect the distribution of habitats and, importantly, species composition [26]. The coastal savannah and forest zones have a bimodal rainfall pattern, allowing for two peaks of malaria transmission, while the Sahel zone has a unimodal rainfall pattern, giving rise to seasonal malaria transmission.

The ecology of larval mosquitoes has implications for vector control, hence the need to understand the productivity and dynamics of larval habitats in the changing environment in order to model and predict the abundance of adult mosquitoes and ultimately develop effective control tools or programmes [2, 27]. The aim of this study, therefore, was to investigate the ecology of *Anopheles* mosquito larvae in different ecological zones in Ghana. The availability of larval habitats, their productivity, and distribution among different zones in Ghana was studied. The spatio-temporal distribution and species composition of larval malaria and lymphatic filariasis vectors were investigated.

## Methods

### Study sites

**Fig. 1:**
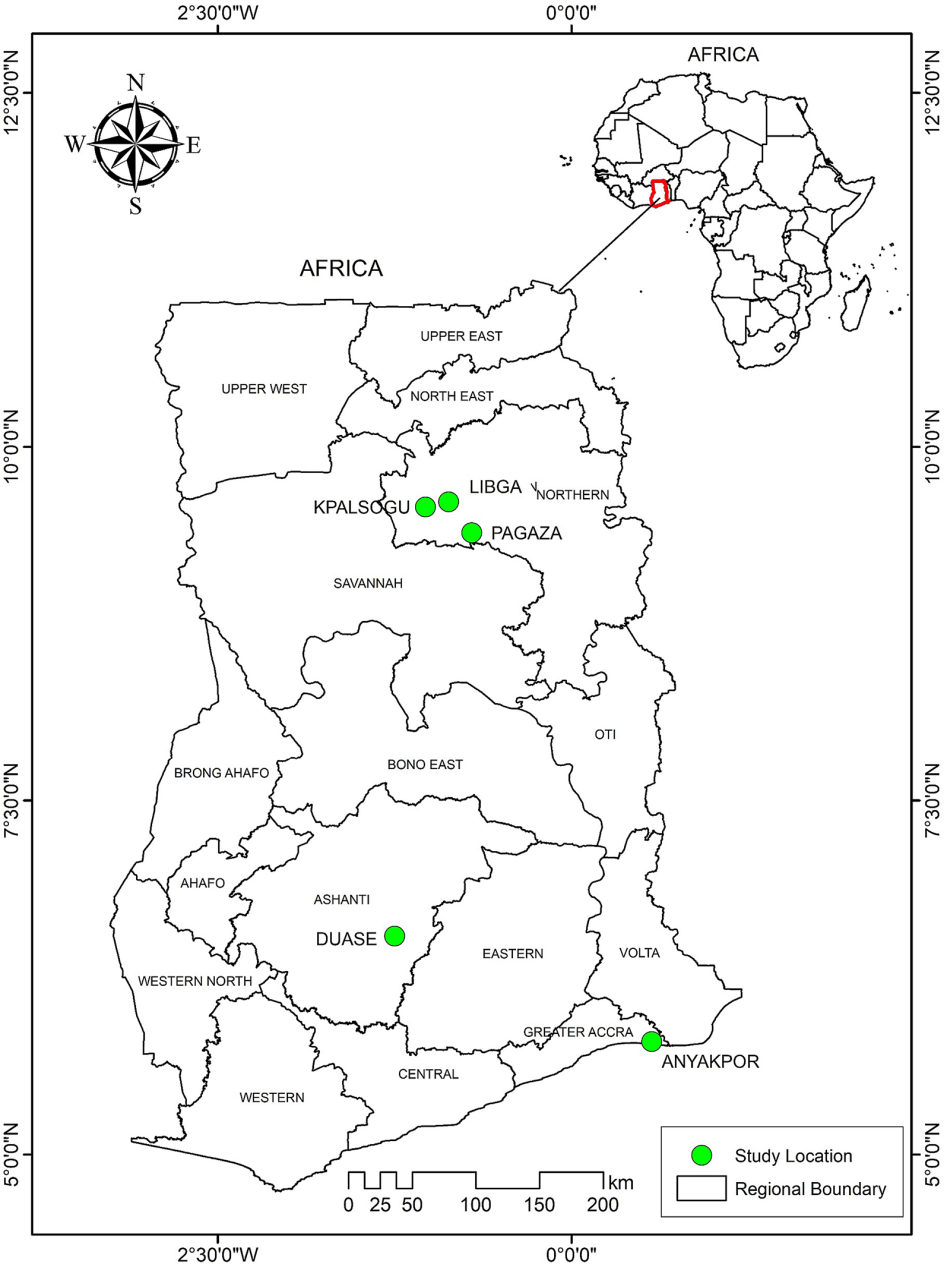
Map of study area

The study was conducted in five locations in three ecological landscapes of Ghana—the coastal savannah, the forest, and the Sahel savannah zones (Fig. 1). Anyakpor (5°46′51.96″N, 0°35′12.84″E) was the site in the coastal savannah area. It is a rural coastal community about 5 km west of Ada Foah in southern Ghana, and has a dry equatorial climate with temperatures ranging from 23 °C to 28 °C throughout the year and maximum temperatures reaching 33 °C. Its rainfall pattern is bimodal, with a long rainy season from April to June and a short rainy season from September to November. It has coastal savannah type vegetation. The community is divided into two main parts—the fishing and farming communities. The farmers are involved in vegetable farming on raised beds. Because it is a low-lying area with a high water table, farmers in Anyakpor in coastal Ghana dig wells and ponds to get underground water for irrigation purposes. The nature of the soil of the communities along the coast makes it difficult to hold water; as a result, there are little to no breeding habitats within the settlement areas of the community throughout the year. *An. gambiae* s.l. is the dominant malaria vector in the coastal savannah zone, followed by *An. funestus* [28].

Duase (6°32′3.05″N, 1°14′42.22″W) was the site located in the forest zone. It is a rural community close to Konongo. It has a wet semi-equatorial climate characterized by two distinct rainy seasons, with a long rainy season from May to July and a short rainy season from September to November. The mean annual temperature and relative humidity are 26 °C and 77%, respectively. The vegetation is a semi-deciduous forest composed of open and closed forests. Duase is drained by one main river which stretches throughout the community, forming large and small water collections at various points which serve as suitable breeding habitats for mosquitoes. The river is diverted at several points to create ponds and wells for farm animals as well as for irrigation purposes. *An. gambiae* is the major malaria vector in the forest area.

Kpalsogu (9°33′45.2″N, 1°01′54.6″W), Pagaza (9°22′33.34″N, 0°42′29.67″W), and Libga (9°35′32.26″N, 0°50′48.8″W) were the selected sites in the Sahel savannah region of northern Ghana. They have a unimodal rainfall pattern from May to November. The mean annual temperature, which is 28 °C, appears to be favourable for *Anopheles* larval development, but temperature can reach a maximum of 42 °C. There are dug-out dams and other water impoundments which collect water during the rainy season for irrigation in the dry season. In the rainy season, these dams overflow, creating many swamps which are suitable breeding habitats for *Anopheles* mosquitoes. Water from the dams which is diverted through canals to farms also serves as breeding sites for mosquitoes. In the harsh dry season, most of these dug-out dams dry up, forming small, temporary open sunlit water collections which are suitable breeding habitats for *An. gambiae* s.s. [29]. *An. gambiae* s.l. and *An. funestus* are the major malaria vectors in the Sahel savanna zone. Secondary vectors found in the Sahel savannah include *An. rufipes*, *An. nili*, and *An. pharoensis* [29], Kpalsogu has been an active IRS site since 2008; in Libga, IRS was stopped after 2014, and Pagaza has never been under IRS intervention. This study was undertaken during the dry (February–March) and rainy (May in the forest zone and coastal savannah zone, and August–September in the Sahel savannah zone) seasons of 2019. Each habitat was sampled once during each season.

### Larval habitat characterization

All larval habitats in each site were classified as natural or man-made. Natural habitats included swamps, streams, and natural ponds, while man-made habitats included drainage ditches, footprints, and hoofprints. Land-use type was classified based on the natural vegetation and activities taking place on the land where the larval habitat was found. These included forest for sites with high canopy cover, farmland for cultivated areas, pasture for grazing areas, shrubland for bushy areas with short trees, roads, and swamps, and compounds or home for places with human settlement. The length and width of each habitat were measured and recorded in metres. The percentage of vegetation covering the surface of the water was visually estimated [30]. The vegetation cover was categorized as follows: zero if vegetation was not present in the habitat, ≤ 24% surface coverage, and 25–49, 50–74, and 75–100% surface coverage [31].

### Larval sampling and densities

Larval sampling was conducted for all potential breeding sites by the standard dipping method using the WHO 350 ml standard dipper. The size of each habitat was categorized as ≤ 1, > 1–2, > 2–5, > 5–10, > 10–100, or > 100 m, and a maximum of 2, 4, 6, 10, 50, and 150 dips were taken, respectively (i.e., depending on the size of the habitat), as described by Gouagna and Mereta [28, 36]. For habitats pf much smaller size such as hoofprints and footprints, a ladle was used to collect the samples. Larvae collected were classified as early instars (L1 and L2) or late instars (L3 and L4). The number of larvae and pupae were recorded, and the larval density was estimated as the ratio of the number of larvae collected per dip [32–35].

### Mosquito species identification

Anopheline larvae specimens were transported to the insectary of the Department of Medical Microbiology, University of Ghana, where they were bred into adults. The larvae were fed on TetraMin^®^ fish meal and maintained at 27 ± 2 °C. Emerging adult mosquitoes were morphologically identified under a stereomicroscope using the taxonomic keys by Gillies and Coetzee [36]. *Anopheles gambiae* s.l. were further identified to sibling species and molecular form using rDNA polymerase chain reaction (PCR) [37] and PCR–restriction fragment length polymorphism (RFLP) [38] analysis, respectively.

### Data analysis

Descriptive analysis was performed to compare the abundance of the various habitat types and larval densities in the different study sites (ecological zones) and seasons. Larval densities were calculated by dividing the total number of larvae collected by the total number of dips taken. The total number of dips for smaller habitats such as footprints and hoofprints was considered to be one dip. A test for normality of the larval density distribution using the Shapiro–Wilk test showed a non-normal distribution. The density of *Anopheles* mosquito larvae was compared among the various breeding habitats and study sites. The Mann–Whitney *U* test and the Kruskal–Wallis test were used to test the associations between continuous and categorical variables. The chi-square and Fisher's exact tests were used to test the association between two categorical variables. Logistic regression was used to assess the association between the habitat characteristics with categorical data and the presence of *Anopheles* larvae. Nested generalized linear mixed models with sites nested within ecological zones were used to model the effect of habitat characteristics on larval densities. All statistical analyses were conducted in STATA version 15 software (StataCorp. 2017. Stata Statistical Software: Release 15. College Station, TX: StataCorp LLC).

## Results

### Distribution and abundance of larval habitats in different ecological zones and seasons

**Fig. 2:**
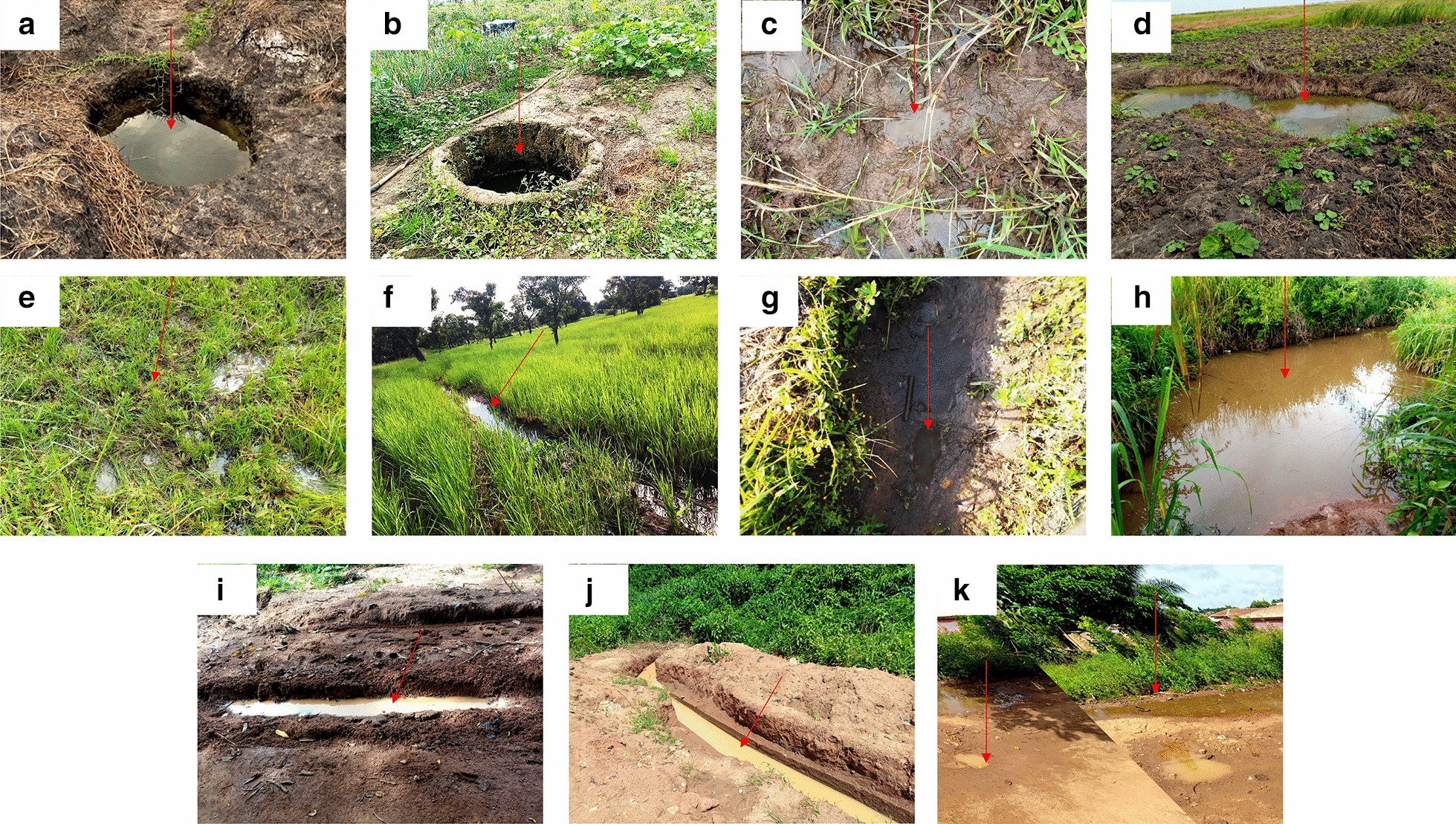
Habitat types found during the study period. **a** Dug-out well, **b** concrete well, **c** hoofprints, **d** man-made pond, **e** swamp, **f** furrow, **g** footprint, **h** natural pond, **i** drainage ditch, **j** tyre track, **k** puddle

A total of 383 breeding habitats comprising 11 different habitat types were encountered and recorded during the study (Fig. 2). Most of the habitats were man-made (69.71%, 267/383), and the others (30.29%, 116/383) were natural. The most abundant habitat type was man-made ponds (27.15%, 104/383), which were present throughout the rainy and dry seasons mostly on farmlands. This was followed by natural ponds (12.01%, 46/383), swamps (11.49%, 44/383), dug-out wells (10.97%, 42/383), and concrete wells (9.92%, 38/383). Other habitat types included tyre tracks (7.83%, 30/383), puddles (6.27%, 24/383), and drainage ditches (6.01%, 23/383). The less abundant habitat types were furrows (4.18%, 16/383), hoofprints (2.87%, 11/383), and footprints (1.31%, 5/383) (Table 1).

**Table 1:** Distribution and abundance of larval habitats

	Concrete well	Dug-out well	Natural pond	Man-made pond	Drainage ditch	Tyre track	Footprint	Hoofprint	Swamp	Furrow	Puddle	Total
Dry season												
Anyakpor	5 (62.50)	27 (90.00)	0	22 (95.65)	0	0	0	0	0	0	0	54 (38.57)
Duase	2 (25.00)	3 (10.00)	9 (31.03)	1 (4.35)	4 (22.22)	0	0	0	2 (10.53)	0	5 (100)	26 (18.57)
Kpalsogu	0	0	9 (31.03)	0	2 (11.11)	0	1 (100)	1 (100)	16 (84.21)	1 (16.67)	0	30 (21.43)
Libga	1 (12.50)	0	5 (17.24)	0	9 (50.00)	0	0	0	0	5 (83.33)	0	20 (14.29)
Pagaza	0	0	6 (20.69)	0	3 (16.67)	0	0	0	1 (5.26)	0	0	10 (7.14)
Total	8 (100)	30 (100)	29 (100)	23(100)	18 (100)	0	1 (100)	1 (100)	19 (100)	6 (100)	5 (100)	140 (100)
Rainy season												
Anyakpor	30 (100)	8 (66.67)	1 (5.88)	75 (92.59)	0	0	0	0	0	0	0	114 (46.91)
Duase	0	4 (33.33)	9 (52.94)	6 (7.41)	3 (60)	8 (26.67)	0	0	2 (8)	0	4 (21.05)	36 (14.81)
Kpalsogu	0	0	2 (11.76)	0	0	21 (70)	3 (75)	10 (100)	14 (56)	6 (60)	10 (52.63)	66 (27.16)
Libga	0	0	2 (11.76)	0	0	0	0	0	6 (24)	4 (40)	0	12 (4.94)
Pagaza	0	0	3 (17.65)	0	2 (40)	1 (3.33)	1 (25)	0	3 (12)	0	5 (26.32)	15 (6.17)
Total	30 (100)	12 (100)	17 (100)	81 (100)	5 (100)	30 (100)	4 (100)	10 (100)	25 (100)	10 (100)	13 (100)	243 (100)

Numbers in parenthesis are percentages

The distribution of mosquito larval habitat types and their abundance varied among the study sites (*χ*^2^ = 498.2658; df = 40; *p* = 0.0001) as well as ecological zones (*χ*^2^ = 369.5865; df = 20; *p* = 0.0001) (Additional file 1: Table S1). In Anyakpor in the coastal savanna zone, only four habitats types were encountered: man-made ponds (57%, 97/168), dug-out wells (20.83%, 35/168), concrete wells (20.83%, 35/168), and a natural pond (0.60%, 1/168). In Duase in the forest area, eight habitat types were found: natural ponds (29.03%, 18/62), puddles (14.52%, 9/62), tyre tracks (12.90%, 8/62), man-made ponds (11.29%, 7/62), drainage ditches (11.29%, 7/62), dug-out wells (11.29%, 7/62), swamps (6.455, 4/62), and concrete wells (3.23%, 2/62). Eight habitat types were also encountered in Kpalsogu in the Sahel savannah area. These included swamps (31.50%, 30/96), tyre tracks (21.88%, 21/96), hoofprints (11.46, 11/96), natural ponds (11.46, 11/96), puddles (10.42%, 10/96), furrows (7.29%, 7/96), footprints (4.17%, 4/96), and drainage ditches (2.08%, 2/96). In Libga in the Sahel savannah area, only four habitat types were found: furrows (28.13%, 9/32), drainage ditches (28.13%, 9/32), natural ponds (21.88%, 7/32), and a concrete well (3.13%, 1/32). In Pagaza, also in the Sahel savannah area, the six habitats encountered were natural ponds (36%, 9/25), puddles (20%, 5/25), drainage ditches (20%, 5/25), swamps (16%, 4/25), tyre tracks (4%, 1/25), and footprints (4%, 1/25).

Larval habitats were significantly more abundant in the rainy season (63.45%, 243/383) than in the dry season (36.55%, 140/383) (*χ*^2^ = 91.3295; df = 10; *p* = 0.001) (Additional file 1: Table S1). Dug-out wells were the most abundant habitat type during the dry season, while man-made ponds were the most abundant during the rainy season. The presence of a larval habitat type was associated with the land-use type (*χ*^2^ = 587.4192; df = 60; *p* = 0.0001). Larval habitats were mostly found on farmlands (58.75%), followed by pastures (16.19%) and on roads (13.05%). The rest were present in forested areas (4.70%), around homes or compounds (4.18%), by streams and rivers (1.83%), and in swamps (1.31%). Most of the habitats had vegetation cover of less than 24% (*χ*^2^ = 215.1340; df = 40; *p* = 0.0001) (Additional file 1: Table S1).

### Larval habitat types, and the presence and densities of *Anopheles* larvae

The presence of *Anopheles* larvae was dependent on the type of habitat present (*χ*^2^ = 41. 3651; df = 10; *p* < 0.0001). Even though there was a significant increase in the number of habitats during the rainy season (*χ*^2^ = 91.3295; df = 10; *p* < 0.0001) (Additional file 1: Table S1) compared to the dry season, the proportion of habitats where *Anopheles* larvae were present did not differ significantly between the two seasons (*χ*^2^ = 0.0051; df = 1; *p* = 0.943) (Additional file 2: Table S2). In the dry season, 29.29% (41/140) of the habitats had *Anopheles* larvae present, while in the rainy season, 29.63% (72/243) of the habitats were positive for *Anopheles* larvae. In all, dug-out wells were the most frequent habit of *Anopheles* larvae during the dry season (39.02%, 16/41), followed by man-made ponds (21.95%, 9/41), whereas during the rainy season, man-made ponds were the most commonly inhabited (30.56%, 22/41), followed by swamps (16.67% 12/72) (Table 2).

**Table 2:** Larval habitat types and the presence of larvae during the dry and rainy seasons

Habitat type	Total no. (%) of breeding habitats		No. (%) of habitats with mosquito larvae present		No. (%) of habitats with *Anopheles* spp. present	
	Dry	Rainy	Dry	Rainy	Dry	Rainy
Concrete well	8 (5.71)	30 (12.35)	1 (1.89)	10 (9.09)	0 (0.00)	9 (12.50)
Dug-out well	30 (21.43)	12 (4.94)	18 (33.96)	8 (7.27)	16 (39.02)	6 (8.33)
Natural pond	29 (20.71)	17 (7.00)	7 (13.21)	5 (4.55)	3 (7.32)	3 (4.17)
Man-made pond	23 (16.43)	81 (33.33)	10 (18.87)	47 (42.73)	9 (21.95)	22 (30.56)
Drainage ditch	18 (12.86)	5 (2.06)	7 (13.21)	1 (0.91)	4 (9.76)	1 (1.39)
Puddle	5 (3.57)	19 (7.82)	2 (3.77)	4 (3.64)	2 (4.88)	4 (5.56)
Tyre track	0 (0.00)	30 (12.35)	0 (0.00)	5 (4.55)	0 (0.00)	2 (2.78)
Footprint	1 (0.71)	4 (1.65)	0 (0.00)	3 (2.73)	0 (0.00)	3 (4.17)
Hoofprint	1 (0.71)	10 (4.12)	0 (0.00)	4 (3.64)	0 (0.00)	2 (2.78)
Swamp	19 (13.57)	25 (10.29)	4 (7.55)	15 (13.64)	4 (9.76)	12 (16.67)
Furrow	6 (4.29)	10 (4.12)	4 (7.55)	8 (7.27)	3 (7.32)	8 (11.11)
Total	140 (100)	243 (100)	53 (100)	110 (100)	41 (100)	72 (100)

Numbers in parenthesis are percentages

The type of habitat present was associated with *An. gambiae* s.l. larval density (*χ*^2^ = 29.593; df = 10;* p* < 0.001) (Additional file 2: Table S2). Even though the mean *An. gambiae* s.l. larval density in the rainy season (1.49 larvae/dip) was slightly higher than that in the dry season (1.15 larvae/dip), the difference was not significant (*z* = −0.232; *p* = 0.8166). In Anyakpor, the most productive habitat types were dug-out wells during both the dry (1.6 larvae/dip) and rainy (11.28 larvae/dip) seasons (Table 3). The preferred breeding habitats in Kpalsogu were natural ponds during the dry season (0.89 larvae/dip) and swamps (2.57 larvae/dip) during the rainy season. In Libga, drainage ditches were the only habitat type present in the dry season, with mean larval density of 0.3 larvae/dip, while furrows were the only productive habitats in the rainy season (1.83 larvae/dip). There were no productive habitats in Pagaza during the dry season, but in the rainy season the most productive habitat type was puddles (1.44 larvae/dip). In Duase, the most productive habitat type was dug-out wells in both the dry season (1.47 larvae/dip) and the rainy season (2.05 larvae/dip) (Table 3).

**Table 3:** *Anopheles* larval density in the dry and rainy seasons

Habitat type	Larval density (larvae/dip)									
	Anyakpor		Duase		Kpalsogu		Libga		Pagaza	
	Dry	Rainy	Dry	Rainy	Dry	Rainy	Dry	Rainy	Dry	Rainy
Concrete well	0	1.23	0	0	0	0	0	0	0	0
Dug-out well	1.59	11.28	1.47	2.05	0	0	0	0	0	0
Natural pond	0	0	0.09	0.03	0.89	0	0	0	0	0.15
Man-made pond	0.56	0.84	1.20	0.97	0	0	0	0	0	0
Puddle	0	0	0.44	0	0	0.25	0	0	0	1.44
Drainage ditch	0	0	0.55	0.25	0	0	0.30	0	0	0
Tyre track	0	0	0	0.9	0	0	0	0	0	0
Furrow	0	0	0	0	0	2.35	0.21	1.83	0	0
Hoofprint	0	0	0	0	0	0.46	0	0	0	0
Footprint	0	0	0	0	0	2.00	0	0	0	0.2
Swamp	0	0	0	0	0.27	2.57	0	1.13	0	0.15

### Abundance and distribution of *Anopheles* larvae in the different ecological zones

A total of 7894 mosquito larvae were collected during this study. Of this number, 2152 (27.26%) were anophelines, while 5742 (72.74%) were culicines. Of the anopheline species, 2128 (98.88%) were *An. gambiae* s.l., 16 (0.74%) were *An. rufipes*, and 8 (0.37%) were *An. pharoensis*. During the rainy season, 1500 (70.49%) *An. gambiae* s.l. were collected, while 628 (29.51%) were collected in the dry season.

Anyakpor in the coastal savannah area had the highest abundance of *An. gambiae* s.l. larvae (1286), with 343 (26.67%) occurring in the dry season and 943 (73.33%) in the rainy season (Table 4). In Duase, situated in the forest area, 30.17% of *An. gambiae* s.l. larvae were collected during the dry season, while 69.83% were collected in the rainy season. Kpalsogu and Libga in the Sahel savannah recorded 39.00 and 35.79% of *An. gambiae* s.l. larvae in the dry season, while the rainy season contributed 61.00 and 64.21%, respectively. In Pagaza, also in the Sahel savannah, *An. gambiae* s.l. larvae were found only in the rainy season (Table 4), with none during the dry season.

**Table 4:** Abundance of *An. gambiae* s.l. larvae during the dry and rainy seasons

Study site	No. (%) of *An. gambiae* s.l.		Total (%)
	Dry season	Rainy season	
Anyakpor	343 (54.62)	943 (62.87)	1286 (60.43)
Duase	54 (8.60)	125 (8.33)	179 (8.41)
Kpalsogu	163 (25.96)	255 (17.00)	418 (19.64)
Libga	68 (10.83)	122 (8.13)	190 (8.93)
Pagaza	0 (0.00)	55 (100)	55 (2.59)
Total	628 (100)	1500 (100)	2128 (100)

The distribution of *An. gambiae* sibling species varied across the ecological zones (*χ*^2^ = 45.9887 df = 8; *p* = 0.0001). *An. coluzzii* was the most abundant species (53.44%) in all ecological zones, followed by *An. gambiae* s.s. (25.98%) and *An. arabiensis* (6.27%), which were found only in the Sahel savannah: Kpalsogu (19.30%), Libga (17.86%), and Pagaza (35.71%). *Anopheles melas* were the least abundant species (4.19%) and were present only in Anyakpor in the coastal savannah area (Table 5). All the species were more abundant in the rainy season (*χ*^2^ = 21.2510; df = 2; *p* = 0.0001) than in the dry season. *An. gambiae* s.s. and *An. coluzzii* were found in all the habitat types encountered in this study. *An. arabiensis* were predominantly found in swamps (52.38%) and furrows (28.57%), whereas *An. melas* were found in dug-out wells (55.56%) and man-made ponds (44.44%). While *An. rufipes* were found only in Kpalsogu and Libga in the Sahel savannah area, *An. pharoensis* were found in Anyakpor in the coastal savannah area and Libga in the Sahel savannah area *An. pharoensis* were found only in man-made ponds (75.00%) and furrows (25.00%). *An. rufipes* were found in swamps (56.25%), footprints (25.00%), furrows (12.50%), and puddles (6.25%).

**Table 5:** Distribution of larval *Anopheles* species in the study sites

*Anopheles* larvae	No. of sites (%)					Total
	Anyakpor	Duase	Kpalsogu	Libga	Pagaza	
*Anopheles* gambiae s.l.						
* An. gambiae* s.s.	265 (20.62)	75 (42.11)	132 (31.58)	75 (39.29)	12 (21.43)	559 (25.98)
* An. coluzzii*	782 (60.82)	104 (57.89)	176 (42.11)	68 (35.71)	20 (35.71)	1150 (53.44)
* An. arabiensis*	0	0	81 (19.30)	34 (17.86)	20 (35.71)	135 (6.27)
* An. melas*	119 (9.28)	0	0	0	0	119 (5.53)
Unidentified *An. gambiae* species	119 (9.28)	0	29 (7.02)	13 (7.14)	4 (7.14)	165 (7.67)
Other anophelines						
* An. pharoensis*	6 (0.46)	0	0	2 (1.03)	0	8 (0.37)
* An. rufipes*	0	0	13 (3.02)	3 (1.54)	0	16 (0.74)
Total	1292 (100)	179 (100)	431 (100)	195 (100)	55 (100)	2152 (100)

### Habitat characteristics, occurrence, and densities of *Anopheles* larvae

The type of habitat influenced the presence of *Anopheles* larvae (*χ*^2^ = 41.3651; df = 10; *p* = 0.0001) and their density (*p* < 0.01). A significant majority (84.07% (95/113) of *Anopheles*-positive habitats were less than 10 m^2^ in size (*χ*^2^ = 11.9217; df = 2; *p* = 0.0001). Land-use type influenced both the presence of *Anopheles* larvae (*χ*^2^ = 26.5920; df = 6; *p* = 0.0001) (Additional file 2: Table S2) and their larval density (*χ*^2^ = 16.117; df = 6; *p* = 0.013) (Additional file 2: Table S2). Fifty-five percent of all larval habitats found around homes or compounds contained *Anopheles* larvae. The odds of finding *Anopheles* larvae in any habitat was twice as high if the vegetation cover was less than 24% (OR 2.24 [1.02, 4.93]; *p* = 0.045) (Table 6). As the vegetation cover increased, the density of *Anopheles* larvae decreased (*B* = −0.016 [−0.28, 0.003]; *p* = 0.015) (Additional file 3: Table S3). The present study, again, showed that *Anopheles* larvae share a habitat preference with culicine larvae. The likelihood of encountering *Anopheles* larvae in a breeding habitat was more than three times as high when culicines were present (OR 3.13 [1.75,5.59]; *p* < 0.01) (Table 6).

**Table 6:** Logistic regression table showing habitat characteristics that influence the presence of *Anopheles* larvae

Characteristic	Category	*Anopheles* present	*Anopheles* absent	Adjusted OR (CI)	*p* value
Habitat type	Concrete well	9/38 (23.68)	29/38 (76.32)	1	
	Dug-out well	22/42 (52.38)	20/42 (47.62)	2.59 (0.87, 7.54)	0.107
	Natural pond	6/46 (13.04)	40/46 (86.96)	0.27 (0.01, 9.27)	0.468
	Man-made pond	31/104 (29.81)	73/104 (70.19)	0.83 (0.30, 2.28)	0.714
	Drainage ditch	5/23 (21.74)	18/23 (78.26)	2.26 (0.23, 21.98)	0.483
	Puddle	6/24 (25.00)	18/24 (75.00)	0.51 (0.02, 17.20)	0.710
	Tyre track	2/30 (6.67)	28/30 (93.33)	6.43 (0.24, 173.44)	0.268
	Footprint	3/5 (60.00)	2/5 (40.00)	24 (1.57, 386.77)	0.023
	Hoofprint	2/11 (18.18)	9/11 (81.82)	2.26 (0.14, 35.30)	0.562
	Swamp	16/44 (36.36)	28/44 (63.64)	1.33 (0.04, 43.59)	0.870
	Furrow	5/16 (31.25)	11/16 (68.75)	8.17 (0.64, 104.94)	0.107
Nature of habitat	Natural	33/116 (28.45)	83/116 (71.55)	1	
	Man-made	80/267 (29.96)	187/267 (70.04)	0.15 (0.01, 2.74)	0.203
Habitat size categorical	< 10 m	95/295 (32.20)	200/295 (67.80)	1	
	10–100 m	18/62 (29.03)	44/62 (70.97)	1.73 (0.74, 4.01)	0.203
	> 100 m	0	26/26 (100)	1	
Vegetation cover categorical	None	21/92 (22.83)	71/92 (77.17)	1	
	< 24%	38/98 (38.78)	60/98 (61.22)	2.24 (1.02, 4.93)	0.045
	25–49%	14/44 (31.82)	30/44 (68.18)	2.62 (0.50, 13.74)	0.253
	50–74%	16/47 (34.04)	31/47 (65.96)	3.06 (0.20, 46.04)	0.419
	75–100%	74/95 (77.89)	21/95 (22.11)	2.61 (0.07, 101.11)	0.607
Land-use type	Farmland	72/225 (32.00)	153/225 (68.00)	1	
	Pasture	23/62 (37.10)	39/62 (62.90)	0.82 (0.29, 2.33)	0.716
	River/stream	0/7	7/7 (100)	1	
	Swamp	0/5	5/5 (100)	1	
	Road	3/50 (6.00)	47/50 (94.00)	0.10 (0.01, 0.95)	0.045
	Compound/home	10/18 (55.56)	8/18 (44.44)	3.06 (1.00, 9.36)	0.050
	Forest	5/16 (31.25)	11/16 (68.75)	1.05 (0.24, 4.55)	0.943
Season	Dry	41/140 (29.29)	99/140 (70.71)		
	Rainy	72/243 (29.63)	171/243 (70.37)	1.55 (0.83, 2.92)	0.171
Presence of culicines	Absent	59/279 (21.15)	220/279 (78.85)	1	
	Present	54/168 (32.14)	114/168 (67.86)	3.61 (2.00, 6.53)	0.0001

## Discussion

Understanding the ecology of anopheline larvae in a changing environment is crucial for the development and successful implementation of targeted control measures [36, 37] to supplement current adult vector control tools. In this study, the distribution of *Anopheles* breeding habitats in rural communities in the different ecological zones of Ghana was characterized. The study revealed differences in the abundance and distribution of *Anopheles* breeding habitats in the different ecological zones. Although man-made ponds were the most abundant habitat type, dug-out wells were the most productive for *Anopheles* mosquito larvae. *Anopheles* larvae also preferred to breed in small habitats, while increasing vegetation cover reduced *Anopheles* larval densities.

The common habitat types were man-made ponds, natural ponds, drainage ditches, and swamps. Other habitats such as tyre tracks and puddles are usually formed during the rainy season when rainwater collects on untarred roads [35]. Such habitats are temporal. The habitats encountered were mostly associated with anthropogenic activities. This explains why communities that practice irrigation farming, Anyakpor and Kpalsogu, had the highest number of habitats.

Duase and Kpalsogu had the most diverse breeding habitat types, which included 8 of the 11 habitat types encountered. The persistence of breeding habitats during both dry and rainy seasons in the forest and coastal savannah areas accounts for perennial malaria found within these sites, while seasonal variations are observed in the Sahel savannah areas [39, 40].

In all, the most abundant habitat type was man-made ponds. Natural ponds were the only habitat type found in all the study sites. Hoofprints were found only in the Sahel savannah zone where livestock are left to graze on swampy pastures. Most of the habitats were man-made and found on farmlands. This emphasizes the importance of human activities and, for that matter, land-use in the creation of *Anopheles* breeding habitats and the impact they have on malaria transmission.

The fact that most of the breeding habitats are found on farmland can be attributed to the practice of irrigation. Irrigation provides ideal breeding habitats for *Anopheles* vectors, and this study corroborates that of Appawu et al. [41], who found that irrigated fields generated large numbers of mosquitoes. It is important to note that agrochemicals used on these farms end up polluting the water sources which serve as breeding sites on these farms, thereby leading to the development and spread of insecticide resistance by exposing mosquito larvae to high or sublethal doses of the agrochemicals [42–45].

The variation in the presence of *Anopheles* larvae may be due to the differences in the physical, chemical, and biological properties as well as the quality of the water present in the various habitats [46]. These properties directly influence the choice of oviposition sites by gravid females and also influence the development and survivorship of larvae [47–50]. The presence of *Anopheles* larvae in man-made ponds and dug-out wells further establishes the influence of human activities on the presence and distribution of *Anopheles* vectors [31, 37, 50, 53–55].

The presence of *Anopheles* larvae in habitats close to human settlements, where it was easy to find the next blood meal source, corroborates the findings of studies from Kenya [51, 52], which also suggest that because *An. gambiae* s.l. are closely associated with humans, they will make use of the closest habitat for oviposition when they become gravid [51, 52]. Choosing habitats close to human settlements where *An. gambiae* may have taken a blood meal is also an evolutionary strategy to conserve energy [46, 53].

This study also revealed that *Anopheles* larvae were predominantly present in breeding habitats with vegetation cover of less than 24%. Similar findings have been observed in Kenya [54–56]. Low vegetation cover allows the habitat to be more exposed to sunlight, a preference for ovipositing mosquitoes [5]. Also, adequate exposure to sunlight warms the water to suitable temperatures, as temperature is also a key factor influencing larval development and survival [2, 5, 7, 57]. Inadequate exposure to sunlight caused by high vegetation cover affects the photosynthetic efficiency of algae biomass which serves as food for mosquito larvae [58]. It was evident in this study that as the percentage of vegetation cover increased, the density of *Anopheles* larvae decreased. This is in line with studies conducted in Ethiopia [35, 59]. As vegetation cover increases, the amount of sunlight reaching the habitat is decreased.

In Anyakpor in the coastal zone, the predominant species was *An. coluzzii* (60.82%), similar to what was reported by Kudom [60] and Fossog et al. [61], whose model showed *An. coluzzii* to dominate the coastal line of Africa. The species was mostly found in man-made ponds, dug-out wells, and concrete wells. Compared to *An. gambiae* s.s., both inland and coastal *An. coluzzii* are known to have a higher tolerance to salinity [61, 62]. *An. melas* in Anyakpor were found only in dug-out wells and man-made ponds, because these habitats are fed by salty underground water which *An. melas* prefer to breed in, unlike concrete wells which are fed by rainwater. *An. coluzzii* and *An. gambiae* s.s. were the only species found in Duase, in the forest zone, with *An. coluzzii* being the dominant species, which is contrary to the findings of other studies [39, 63, 64]. This could be as a result of the deforestation which is caused by rapid urbanization. Rapid deforestation affects climatic conditions, and this might be causing Duase to become drier, thereby allowing *An. coluzzii* to thrive there. *An. coluzzii* was also the dominant species in Kpalsogu, followed by *An. gambiae* s.s. and *An*. *arabiensis*. On the other hand, *An. gambiae* s.s. was the dominant species in Libga. In Pagaza, *An. coluzzii* and *An*. *arabiensis* were co-dominant. *An. arabiensis* were found only in the Sahel savannah area because of its dry sub-arid environments [65]. *An.* arabiensis prefer to be zoophilic even though they can also be anthropophilic [66], and this can explain why they were most commonly found in swamps, since swamps in the Sahel savannah areas mostly serve grazing cattle. As shown by this study, *An. coluzzii* and *An. gambiae* s.s. are known to live in sympatry in most parts of Ghana [60]. However, *An. coluzzii* predominates in the coastal and Sahel savannah regions of Ghana [63, 64, 67]. The dominance of *An. coluzzii* in the coastal regions of Ghana has been attributed to permanent habitats created on irrigation farms [63, 64]. *An. coluzzii* is usually seen breeding in large permanent habitats, but in this study they were also found in small temporary habitats such as hoofprints. A study by Edillo et al. [68] showed that *An. coluzzii* preferred different breeding habitat types from *An. gambiae* s.s. and *An. arabiensis*, but this study showed otherwise. *An. pharoensis* and *An. rufipes* are secondary malaria vectors in Ghana [15], and even though they are less abundant, they can gradually replace the current malaria vectors and become dominant in the very near future.

This study might have missed some of the larval habitats and some species of mosquitoes, since it was difficult to access all habitats in all study sites. Some habitats might have been in dense thicket and bushes that were not reached for sampling. This study provides baseline insight into the development of integrated larval source management suitable for the larval habitats in the various study sites. The results revealed that irrigated farms contribute to higher populations of malaria mosquitoes, as this system of agriculture creates many habitats suitable for *Anopheles* mosquito larvae. As a result, communities that practice irrigation farming have a higher abundance of *Anopheles* larvae. This study, again, revealed that human activities contribute greatly to the presence and abundance of *Anopheles* mosquito larvae. This implies that changes in agricultural methods, including methods of irrigation and environmental management such as surface water drainage, landfilling, and land reclamation, would be extremely beneficial in controlling malaria transmission. Considering that *An. gambiae* s.l. prefers to breed in small habitats, habitat modification would be a suitable method of larval source reduction. Other integrated larval source management approaches such as water and environmental management and biological control methods are also feasible. In communities that rely on the same water collections for domestic purposes, and in situations where the water collections cannot be drained, microbial bio-larvicides may be used.

## Conclusion

In this study, the presence and availability of *Anopheles* breeding habitats varied among the study site, and, ecological zones. The abundance of breeding habitats was influenced by rainfall, as more habitats were created during the rainy season and this, in turn, increased the abundance of *Anopheles* mosquitoes in all the ecological zones. Man-made ponds were the most abundant breeding habitat in the coastal savannah zone, while natural ponds were the most abundant in both the forest and Sahel savannah zones. Dug-out wells were the most productive habitats in the coastal savannah and forest zones, while furrows were the most productive in the Sahel savannah zone. *Anopheles coluzzii* was the predominant species in all the study sites. *Anopheles melas* was found only in the coastal savannah, whereas *An. arabiensis* was found only the Sahel savanna zone. The abundance of *Anopheles* breeding habitats and larvae was influenced by anthropogenic activities. Encouraging people whose activities create the larval habits to become involved in larval source management such as habitat manipulation to stop mosquito breeding will be important for malaria and lymphatic filariasis control.

## Supplementary Information

**Additional file 1: Table S1.** Univariate analysis on the distribution of *Anopheles* breeding habitats.

**Additional file 2: Table S2.** Univariate analysis of habitat characteristics, and the presence and larval density of *Anopheles* larvae.

**Additional file 3: Table S3.** GLMM of habitat characteristics and *Anopheles* larval density.

## Data Availability

All datasets generated and/or analysed during the current study are available on request.
