# Supplementary material for: Larval habitat diversity and Anopheles mosquito species distribution in different ecological zones in Ghana
Source: Parasit Vectors. 2021 Apr 7;14:193. doi: 10.1186/s13071-021-04701-w (PMC8025514; doi:10.1186/s13071-021-04701-w)
Supplement: Supplementary file 1 — Additional file 1: Table S1. Univariate analysis on the distribution of Anopheles breeding habitats. [file 13071_2021_4701_MOESM1_ESM.docx]

**Univariate analysis on the distribution of Anopheles breeding habitats**

**A**

|  | | **Habitat types n/N (%)** | | | | | | | | | | | *p*-value |
| --- | --- | --- | --- | --- | --- | --- | --- | --- | --- | --- | --- | --- | --- |
|  |  | Concrete well | Dugout well | Natural Pond | Man-made Pond | Drainage ditch | Puddle | Tyre track | Footprint | Hoofprint | Swamp | Furrow |  |
| Study Sites | Anyakpor | 35/168 (20.83) | 35/168 (20.83) | 1/168 (0.60) | 97/168 (57.74) | 0 | 0 | 0 | 0 | 0 | 0 | 0 | *p* < 0.0001  ꭓ = 498.2658  df = 40 |
|  | Duase | 2/62 (3.23) | 7/62 (11.29) | 18/62 (29.03) | 7/62 (11.46) | 7/62 (11.46) | 9/62 (14.52) | 8/62 (12.90) | 0 | 0 | 4/62 (6.45) | 0 |  |
|  | Kpalsogu | 0 | 0 | 11/96 (11.46) | 0 | 2/96 (2.08) | 10/96 (10.42) | 21/96 (21.88) | 4/96 (4.17) | 11/96 (11.46) | 30/96 (31.25) | 7/96 (37.29) |  |
|  | Libga | 1/32 (3.13) | 0 | 7/32 (21.88) | 0 | 9/32 (28.13) | 0 | 0 | 0 | 0 | 6/32 (18.75) | 9/32 (28.13) |  |
|  | Pagazaa | 0 | 0 | 9/25 (36.00) | 0 | 5/25 (20.00) | 5/25 (20.00) | 1/25 (4.00) | 1/25 (4.00) | 0 | 4/25 (16.00) | 0 |  |
| Ecological zones | Coastal savannah | 35/168 (20.83) | 35/168 (20.83) | 1/168 (0.60) | 97/168 (57.74) | 0 | 0 | 0 | 0 | 0 | 0 | 0 | *p* < 0.0001  ꭓ = 369.5865  df = 20 |
|  | Forest | 2/62 (3.23) | 7/62 (11.29) | 18/62 (29.03) | 7/62 (11.46) | 7/62 (11.46) | 9/62 (14.52) | 8/62 (12.90) | 0 | 0 | 4/62 (6.45) | 0 |  |
|  | Sahel savannah | 1/153 (0.65) | 0 | 27/153 (17.64) | 0 | 16/153 (10.46) | 15/153 (9.80) | 22/153 (14.38) | 5/153 (3.27) | 11/153 (7.19) | 40/153 (26.14) | 16 (10.46) |  |
| Season | Dry | 8/140 (5.17) | 30/140 (21.43) | 29/140 (20.71) | 23/140 (16.43) | 18/140 (12.86) | 5/140 (3.75) | 0 | 1 (0.71) | 1 (0.71) | 19/140 (13.57) | 6/140 (4.29) | *p* < 0.0001  ꭓ = 91.3295  df = 10 |
|  | Rainy | 30/243 (12.35) | 12/243 (4.94) | 17/243 (7.00) | 81/243 (33.33) | 5/243 (2.06) | 19/243 (7.82) | 30/243 (12.35) | 4/243 (1.65) | 10/243 (4.12) | 25/243 (10.29) | 10/243 (4.12) |  |
| Land-use type | Farmland | 36/225 (16.00) | 38/225 (16.89) | 19/225 (8.44) | 97/225 (43.11) | 9/225 (4.00) | 5/225 (2.22) | 0 | 1/225 (0.44) | 1/225 (0.44) | 10/225 (4.44) | 9/225 (4.00) | *p* < 0.0001  ꭓ = 587.4192  df = 60 |
|  | Pasture | 0 | 0 | 5/62 (8.06) | 0 | 3/62 (4.84) | 4/62 (6.45) | 0 | 4/62 (6.45) | 10/62 (16.13) | 29/62 (46.77) | 7/62 (11.29) |  |
|  | River/stream | 0 | 0 | 1/7 (14.29) | 0 | 5/7 (71.43) | 0 | 0 | 0 | 0 | 1/7 (14.29) | 0 |  |
|  | Swamp | 0 | 0 | 5/5 (100) | 0 | 0 | 0 | 0 | 0 | 0 | 0 | 0 |  |
|  | Road | 0 | 0 | 7/50 (14.00) | 0 | 2 (4.00) | 10 (20.00) | 30 (60.00) | 0 | 0 | 1 (2.00) | 0 |  |
|  | Compound | 2/18 (11.11) | 3/18 (16.67) | 3/18 (16.67) | 7/18 (38.89) | 1/18 (5.56) | 2/18 (11.11) | 0 | 0 | 0 | 0 | 0 |  |
|  | Forest | 0 | 1/16 (6.25) | 6/16 (37.50) | 0 | 3/16 (18.75) | 3/16 (18.75) | 0 | 0 | 0 | 3/16 (18.75) | 0 |  |
| Vegetation cover | < 24 % | 30/197 (15.23) | 39/197 (19.80) | 12/197 (6.09) | 46/197 (23.35) | 3/197 (1.52) | 22/197 (11.17) | 28/197 (14.21) | 3/197 (1.52) | 6/197 (3.05) | 4/197 (2.03) | 4/197 (2.03) | *p* < 0.0001  ꭓ = 180.1651  df = 30 |
|  | 25 – 49 % | 3/44 (6.82) | 0 | 9/44 (20.45) | 10/44 (22.73) | 6/44 (13.64) | 1/44 (2.27) | 2/44 (4.55) | 2/44 (4.55) | 2/44 (4.55) | 6/44 (13.64) | 3/44 (6.82) |  |
|  | 25 -74 % | 1/47 (2.13) | 0 | 1/47 (2.13) | 19/47 (40.43) | 8/47 (17.02) | 1/47 (2.13) | 0 | 0 | 0 | 12/47 (25.53) | 5/47 (10.64) |  |
|  | 75 – 100 % | 4/95 (4.21) | 3/95 (3.16) | 24/95 (25.26) | 29/95 (30.53) | 6/95 (6.32) | 0 | 0 | 0 | 3/95 (3.16) | 22/95 (23.16) | 4/95 (4.21) |  |

**B**

|  |  | Number of Habitats (%) | *p*-value |
| --- | --- | --- | --- |
| Study Sites | Anyakpor | 168/383 (43.86) | *p* < 0.0001  ꭓ = 498.2658  df = 40 |
|  | Duase | 62/383 (16.19) |  |
|  | Kpalsogu | 96/383 (25.07) |  |
|  | Libga | 32/383 (8.36) |  |
|  | Pagazaa | 25/383 (6.53) |  |
| Season | Dry | 140/383 (36.55) | *p* < 0.0001  ꭓ = 91.3295  df = 10 |
|  | Rainy | 243/383 (63.45) |  |
| Land-use type | Farmland | 225/383 (58.75) | *p* < 0.0001  ꭓ = 587.4192  df = 60 |
|  | Pasture | 62/383 (16.19) |  |
|  | River/stream | 7/383 (1.83) |  |
|  | Swamp | 5/383 (1.31) |  |
|  | Road | 50/383 (13.05) |  |
|  | Compound | 18/383 (4.70) |  |
|  | Forest | 16/383 (4.18) |  |
| Vegetation cover | < 24 % | 197/383 (51.44) | *p* < 0.0001  ꭓ = 180.1651  df = 30 |
|  | 25 – 49 % | 44/383 (11.49) |  |
|  | 25 -74 % | 47/383 (12.27) |  |
|  | 75 – 100 % | 95/383 (24.80) |  |
