# Supplementary material for: Larval habitat diversity and Anopheles mosquito species distribution in different ecological zones in Ghana
Source: Parasit Vectors. 2021 Apr 7;14:193. doi: 10.1186/s13071-021-04701-w (PMC8025514; doi:10.1186/s13071-021-04701-w)
Supplement: Supplementary file 2 — Additional file 2: Table S2. Univariate analysis of habitat characteristics, and the presence and larval density of Anopheles larvae. [file 13071_2021_4701_MOESM2_ESM.docx]

## Univariate Analysis of Habitat Characteristics, the Presence, and Larval Density of *Anopheles* larvae

| **Characteristics** | **Categories** | ***Anopheles* present** | ***p*-value** | **Larval density (gmean (CI))** | ***p*-value** |
| --- | --- | --- | --- | --- | --- |
| Habitat type, n/N (%) | Concrete well | 9/38 (23.68) | *p* < 0.0001  ꭓ^2^ = 41.3651  d.f. = 10 | 1.85 (0.52, 6.57) | p = 0.0009  ꭓ^2^ = 29.750  d.f =10 |
|  | Dugout well | 22/42 (52.38) |  | 3.18 (1.80, 5.60) |  |
|  | Natural pond | 6/46 (13.04) |  | 0.45 (0.09, 2.29) |  |
|  | Man-made pond | 31/104 (29.81) |  | 1.01 (0.65, 1.59) |  |
|  | Drainage ditch | 5/23 (21.74) |  | 0.78 (0.22, 2.84) |  |
|  | Puddle | 6/24 (25.00) |  | 1.01 (0.26, 4.00) |  |
|  | Tyre track | 2/30 (6.67) |  | 2.99 (0.00, 8563.95) |  |
|  | Footprint | 3/5 (60.00) |  | 1.69 (0.02, 170.33) |  |
|  | Hoof print | 2/11 (18.18) |  | 1.96 (0.001, 3063.93) |  |
|  | Swamp | 16/44 (36.36) |  | 1.36 (0.75, 2.48) |  |
|  | Furrow | 5/16 (31.25) |  | 1.29 (0.63, 2.67) |  |
| Land-use Type  n/N (%) | Farmland | 72/225 (32.00) | *p* < 0.0001  ꭓ^2^ = 26.5920  d.f.= 6 | 1.45 (1.05, 2.02) | p = 0.0131  ꭓ^2^ = 16.117  d.f. = 6 |
|  | Pasture | 23/62 (37.10) |  | 1.60 (0.95, 2.70) |  |
|  | River/stream | 0/7 |  | - |  |
|  | Swamp | 0/5 |  | - |  |
|  | Road | 3/50 (6.00) |  | 1.89 (0.15, 23.51) |  |
|  | Compound/home | 10/18 (55.56) |  | 1.12 (0.49, 2.57) |  |
|  | Forest | 5/16 (31.25) |  | 0.60 (0.14, 2.63) |  |
| Season  n/N (%) | Dry | 41/140 (29.29) | *p* = 0.943  ꭓ^2^ = 0.0051  d.f. = 1 | 1.15 (0.81, 1.62) | p = 0.7863  z = -0.271 |
|  | Wet | 72/243 (29.63) |  | 1.57 (1.13, 2.20) |  |
| Study site  n/N (%) | Anyakpor | 54/168 (32.14) | *p* = 0.237  ꭓ = 5.6001  d.f.= 4 | 1.68 (1.13, 2.49) | p = 0.4611  χ^2^ = 3.612  d.f = 4 |
|  | Duase | 18/62 (29.03) |  | 0.95 (0.51, 1.78) |  |
|  | Kpalsogu | 24/96 (25.00) |  | 1.70 (1.05, 2.76) |  |
|  | Libga | 13/32 (40.63) |  | 0.99 (0.54, 1.78) |  |
|  | Pagaza | 4/25 (16.00) |  | 0.73 (0.06, 8.92) |  |
| Presence of Culicines | Present | 54/104 (51.92) | *p* < 0.0001  ꭓ^2^ = 34.5002  d.f.=1 | 1.46 (1.02, 2.10) |  |
|  | Absent | 59/279 (21.15) |  | 1.34 (0.95, 1.89) | p < 0.0001  *z = -5.631* |
| Habitat size | < 10 m | 95/295 (32.20) | *p* < 0.0001  ꭓ^2^ = 11.9217  d.f.= 2 | 1.47 (1.12, 1.94) | p = 0.0233  ꭓ^2^ = 7.520  d.f. = 2 |
|  | 10 – 100 m | 18/62 (29.03) |  | 1.09 (0.62, 1.92) |  |
|  | > 100 m | 0 |  | 0 |  |
| Vegetation cover | None | 21/92 (22.83) | *p* = 0.077  ꭓ^2^ = 8.4470  d.f. = 4 | 1.83 (0.95, 3.52) | p = 0.2231  ꭓ^2^ = 5.695  d.f. = 4 |
|  | < 24 % | 38/98 (38.78) |  | 1.89 (1.18, 3.04) |  |
|  | 25 – 49 % | 14/44 (31.82) |  | 1.22 (0.66, 2.27) |  |
|  | 25 -74 % | 16/47 (34.04) |  | 0.63 (0.38, 1.04) |  |
|  | 75 – 100 % | 74/95 (77.89) |  | 1.21 (0.78, 1.87) |  |
