# Supplementary material for: Larval habitat diversity and Anopheles mosquito species distribution in different ecological zones in Ghana
Source: Parasit Vectors. 2021 Apr 7;14:193. doi: 10.1186/s13071-021-04701-w (PMC8025514; doi:10.1186/s13071-021-04701-w)
Supplement: Supplementary file 3 — Additional file 3: Table S3. GLMM of habitat characteristics and Anopheles larval density. [file 13071_2021_4701_MOESM3_ESM.docx]

## GLMM of Habitat Characteristics and *Anopheles* Larval Density

| **Characteristics** | **Categories** | **Unadjusted B (CI)** | ***p*-value** | **Adjusted B (CI)** | ***p*-value** |
| --- | --- | --- | --- | --- | --- |
| Habitat type | Concrete well | 1 |  | 1 |  |
|  | Dugout well | 2.50 (0.99, 4.09) | 0.002 | 2.17 (0.55, 3.78) | 0.009 |
|  | Natural pond | -0.76 (-2.32, 0.79) | 0.337 | 0.22 (-1.75, 2.21) | 0.823 |
|  | Man-made pond | -0.17 (-1.52, 1.17) | 0.804 | 0.11 (-1.29, 1.51) | 0.879 |
|  | Drainage ditch | -0.73 (-2.60, 1.15) | 0.448 | -0.08 (-2.21, 2.06) | 0.943 |
|  | Puddle | -0.47 (-2.32, 1.38) | 0.618 | -0.27 (-2.37, 1.82) | 0.797 |
|  | Tyre track | -0.73 (-2.45, 1.00) | 0.409 | -0.14 (-2.37, 1.82) | 0.918 |
|  | Footprint | 1.06 (-2.31, 4.45) | 0.535 | 0.81 (-2.73, 1.82) | 0.654 |
|  | Hoof print | -0.55 (-2.99, 1.88) | 0.656 | -0.62 (-3.41, 2.17) | 0.692 |
|  | Swamp | 0.13 (-1.44, 1.70) | 0.871 | 0.72 (-1.35, 2.80) | 0.493 |
|  | Furrow | 0.47 (-1.65, 2.59) | 0.664 | 0.61 (-1.64, 2.87) | 0.593 |
| Habitat size | < 10 m | 1 |  | 1 |  |
|  | 10 – 100 m | -0.64 (-1.66, 0.38) | 0.219 | -0.23 (-1.36, 0.89) | 0.684 |
|  | > 100 m | -1.19 (-2.69, 0.30) | 0.117 | -0.10 (-2.01, 1.82) | 0.921 |
| Land-use type | Farmland | 1 |  | 1 |  |
|  | Pasture | 0.02 (-1.02, 1.07) | 0.965 | 0.44 (-1.04, 1.93) | 0.557 |
|  | River/stream | -1.22 (-4.12, 4.57) | 0.391 | -0.24 (-3.30, 2.81) | 0.879 |
|  | Swamp | -1.22 (-4.52, 2.07) | 0.466 | -1.12 (-4.71, 2.48) | 0.542 |
|  | Road | -1.06 (-2.21, 0.07) | 0.067 | -0.72 (-2.72, 1.27) | 0.477 |
|  | Compound/ home | -0.09 (1.88, 1.69) | 0.919 | -0.27 (-2.03, 1.48) | 0.795 |
|  | Forest | -0.91 (-2.80, 0.98) | 0.344 | -0.72 (-2.74, 1.29) | 0.480 |
| Vegetation cover | | -0.01 (-0.25, -0.00) | 0.006 | -0.016 (-0.28, -0.003) | 0.015 |
| Culicine presence | Absent | 1 |  | 1 |  |
|  | Present | 0.91 (0.07, 1.75) | 0.033 | 0.73 (-0.16, 1.62) | 0.106 |
